# Core–shell hydrogel microcapsules enable formation of human pluripotent stem cell spheroids and their cultivation in a stirred bioreactor

**DOI:** 10.1038/s41598-021-85786-2

**Published:** 2021-03-30

**Authors:** Pouria Fattahi, Ali Rahimian, Michael Q. Slama, Kihak Gwon, Alan M. Gonzalez-Suarez, Jadon Wolf, Harihara Baskaran, Caden D. Duffy, Gulnaz Stybayeva, Quinn P. Peterson, Alexander Revzin

**Affiliations:** 1 Department of Physiology and Biomedical Engineering, Mayo Clinic, Rochester, MN 55902 USA; 2 Department of Chemical and Biomolecular Engineering, Case Western Reserve University, Cleveland, OH 44106 USA

**Keywords:** Biological techniques, Biotechnology

## Abstract

Cellular therapies based on human pluripotent stem cells (hPSCs) offer considerable promise for treating numerous diseases including diabetes and end stage liver failure. Stem cell spheroids may be cultured in stirred bioreactors to scale up cell production to cell numbers relevant for use in humans. Despite significant progress in bioreactor culture of stem cells, areas for improvement remain. In this study, we demonstrate that microfluidic encapsulation of hPSCs and formation of spheroids. A co-axial droplet microfluidic device was used to fabricate 400 μm diameter capsules with a poly(ethylene glycol) hydrogel shell and an aqueous core. Spheroid formation was demonstrated for three hPSC lines to highlight broad utility of this encapsulation technology. In-capsule differentiation of stem cell spheroids into pancreatic β-cells in suspension culture was also demonstrated.

## Introduction

There is considerable interest in spheroid cultures of human pluripotent stem cells (hPSCs). This interest is driven in part by the emerging notion that 3D or spheroid cultures are more physiological and better recapitulate signaling present in vivo^1,2^. For example, embryoid bodies, three-dimensional aggregate of cells in early developmental stages, improve differentiation to mature cell types. More recently, the use of spheroids of pluripotent cells has gained popularity for expansion of hPSCs because the 3D cell clusters recapitulate dimensions and gradients of signals present in an early stage embryo^3^.

Another driver for the emergence of spheroid cultures of hPSCs is the need to scale-up. Clinical applications of cellular therapies are likely to require millions to billions of cells (depending on the application)—numbers that are difficult to achieve using standard 2D cultures systems where cell number is proportional to the adherent area. In contrast, scale-up is more feasible in suspension cultures where the number of cells is proportional to the volume of the culture system. As a result, suspension cultures have gained popularity as the method of choice for scalable expansion^4–6^ and differentiation^7,8^ of hPSCs. Given the importance of embryoid body-like geometry for proliferation and differentiation of hPSCs, these cells have typically been formed into spheroids for suspension culture^2^. Spheroid formation in standard suspension cultures occur due to random adhesive interactions between single cells inoculated into the bioreactor (or culture flask) with spheroid assembly being a function of both cell concentration and the speed of rotation/agitation^9^. Such hydrodynamically assembled stem cell spheroids have been utilized widely and with considerable success for both stem cell expansion and for differentiation into a number of adult cell types including hepatocytes^10,11^, pancreatic β-cell^12,13^, cardiomyocytes^14^, neurons^15^, and erythroid cells^16^.

Despite their utility, the current strategies for assembly and cultivation of spheroids have several limitations: (1) not all hPSC lines efficiently and uniformly form spheroids in suspension cultures^17^, likely due to differences in expression of cell adhesion molecules, (2) it is difficult to enhance transport of nutrients in stirred suspension cultures without damaging spheroids with higher shear stress^18^, and (3) it is difficult to control spheroid diameter and size distribution in suspension cultures^19^. We believe that challenges mentioned above may be addressed by encapsulating hPSCs inside biomaterial scaffolds suitable for suspension cultures.

A number of reports described the use of biomaterial scaffolds containing hPSC for stem cell expansion and differentiation^20–23^. However, these biomaterials strategies were not designed with suspension cultures in mind. There has been considerable use of microcarriers for cultivation of hPSCs in suspension cultures^24–26^. These microcarriers (e.g. polystyrene microbeads) may be coated with components of the extracellular matrix (ECM) to promote adhesion of hPSCs, thereby ensuring that more hPSC lines may be used for suspension cultures. However, because stem cells reside on the surface of the microcarriers, they remain exposed to shear stress and may be damaged by agitation. Therefore, there is considerable benefit for placing stem cells inside the microcarriers.

Microcapsules or microparticles for cell encapsulation may be fabricated using either syringe-based or microfluidic nozzles and form uniform or complex composition with examples of the latter being core–shell and Janus-type microcapsules^27–31^. Microcapsules of uniform composition (typically hydrogel) have been used widely for encapsulation of preformed cell aggregates or single cells capable of rapid proliferation^28,32^.

Our team has demonstrated cultivation and differentiation of mouse embryonic stem cells (mESCs) in hydrogel microparticles^32^, however, we were unsuccessful in forming spheroids with encapsulated primary hepatocytes or human ESCs. We reasoned that better outcomes may be attained with microcapsules comprised of a hydrogel shell and an aqueous core where single cells could assemble into spheroids. Microcapsules composed of an alginate shell and aqueous core were demonstrated by He et al. for encapsulation and transplantation of mESCs and cancer stem cells^33,34^. Previously, we employed a similar co-axial flow focusing microfluidic device to fabricate microcapsules with PEG hydrogel shell and aqueous core and demonstrated that non-proliferative primary hepatocytes assembled into spheroids upon encapsulation and remained functional for at least two weeks of culture^35^.

**Figure 1:**
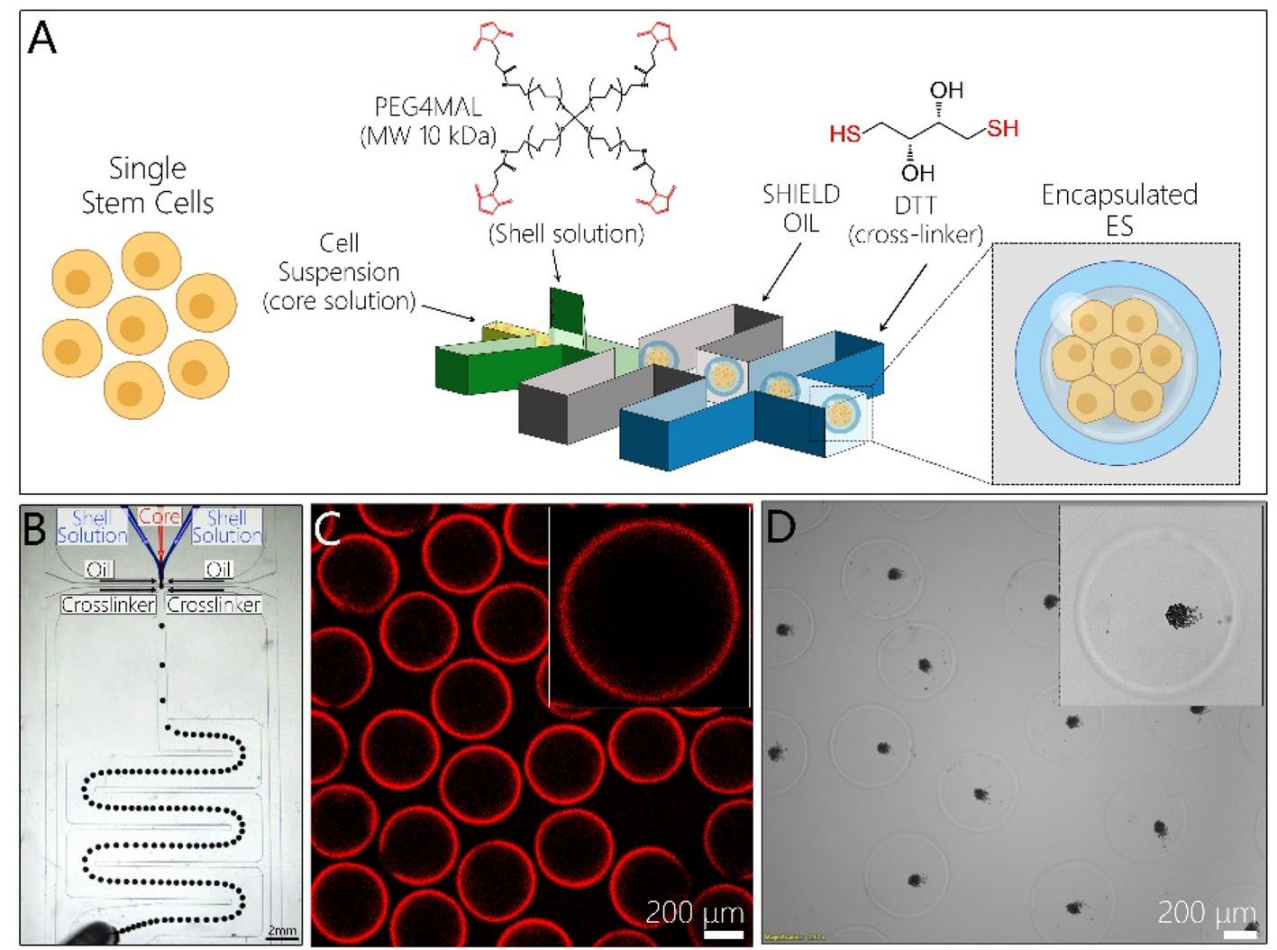
Fabrication of core–shell microcapsules with PEG gel shell and aqueous core. (**A**) Schematic illustration of cell encapsulation process. (**B**) Top view of coaxial flow-focusing device generating a train of aqueous droplets. (**C**) Fluorescence images highlighting that after gellation aqueous droplets became uniformly-sized microcapsules with a thin (5–10 μm shell). Rodamine-labeled PEG was incorporated into the hydrogel shell for visualization purposes. Average microcapsule diameter is 392.6 ± 8.2 μm (n = 109). (**D**) Microbeads included into the core stream during the encapsulation were observed to move freely inside capsules, aggregating in the center of capsules during imaging. This demonstrates that the core of microcapsules was aqueous.

In this report, we assessed the utility of the microcapsules with hydrogel shell and aqueous core for encapsulation of hPSCs (see Fig. 1A). We demonstrated successful encapsulation, spheroid formation and high viability for three different hPSC lines. Using one of the hPSC lines we demonstrated maintenance of pluripotency and successful differentiation of stem cell spheroids into β-cells. In addition, we combined a microfluidic encapsulation with dissociation/filtration module to ensure that ~ 90% of microcapsules contained spheroids. Additional experiments were carried out to demonstrate that microcapsules were protective against shear stress during cultivation in a stirred bioreactor/spinner flask. In summary, our study highlights utility and potential benefits of microfabricated capsules for establishing suspension cultures of hPSCs.

## Results and discussions

### Fabricating microcapsules in a flow focusing microfluidic device

We employed a microfluidic capsule fabrication strategy whereby core and shell streams co-flowed in a co-axial manner and were discretized into droplets by ejection into an oil stream^35^. The shell stream contained molecules of 4-arm PEG-Mal while the core flow stream contained cells, non-reactive PEG and a densifier solution for viscosity matching (see Fig. 1A). Once in the oil phase, the 4-arm PEG-Mal became crosslinked by DTT via “click” chemistry, creating a hydrogel shell region with thickness of ~ 5 μm as measured by fluorescence. Droplet uniformity is a well-known benefit of flow focusing microfluidic devices^36^. As shown in Fig. 1B, our flow focusing microfluidic system generated uniformly sized aqueous droplets which became crosslinked via PEG-Mal/DTT reaction and, upon breaking the emulsion, were collected as uniformly sized hydrogel microcapsules. Core–shell microcapsule has aqueous core with thin hydrogel layer. Analysis of size distribution revealed that for a given set of operational parameters (4 μL min^−1^ core/4 μL min^−1^ shell/50 μL min^−1^ oil flow rates) microcapsule diameters were narrowly distributed around the mean of 392.6 ± 8.2 μm with coefficient of variation of 2.08%. It is worth noting that that the tallest channel in the flow focusing microfluidic device is 300 μm which means that the resultant microparticles should have a diameter of ~ 300 μm and not 400 μm as mentioned above. This discrepancy may be explained by swelling of microparticles upon transfer from oil into aqueous phase. Figure S1 shows that capsules in the oil phase were indeed 300 μm in diameter and that the diameter increased to ~ 400 μm after 2 h in the aqueous phase. Dimensions of capsules are the function of flow rate and microfluidic channel size. 400 µm diameter capsules were chosen to accommodate hPSC spheroids that were typically in the 100 to 200 µm diameter range (see “Discussion” below).

We carried out experiments to confirm core–shell structure of the microcapsules. As shown in Fig. 1C,D PEG-TRITC present in the shell flow stream became incorporated into the hydrogel shell of a microcapsule. Microbeads present in the core flow stream were entrapped inside the microcapsule core. Importantly, as shown in Fig. 1D, microbeads sedimented and aggregated, indicating that the core was aqueous.

As the next step, we wanted to assess viability and spheroid formation for hPSCs encapsulated in our microfluidic devices. To show broad applicability, we chose to encapsulate two human embryonic stem cells (hESCs) lines (HUES-8 and H9) and one induced pluripotent stem cell (iPSC) line (1016). We note that formation of spheroid cultures in a stirred bioreactor/spinner flask is typically a function of cell concentration, stir rate and intrinsic adhesion properties of stem cells (likely related to expression of cell adhesion molecules). HUES-8 cells have been shown to assemble into spheroids at the concentration of 5 × 10^5^ cells mL^−1^ with stirring speed of 70 rpm and have been used by us previously for pancreatic β-cell differentiation in suspension cultures^37^.

**Figure 2:**
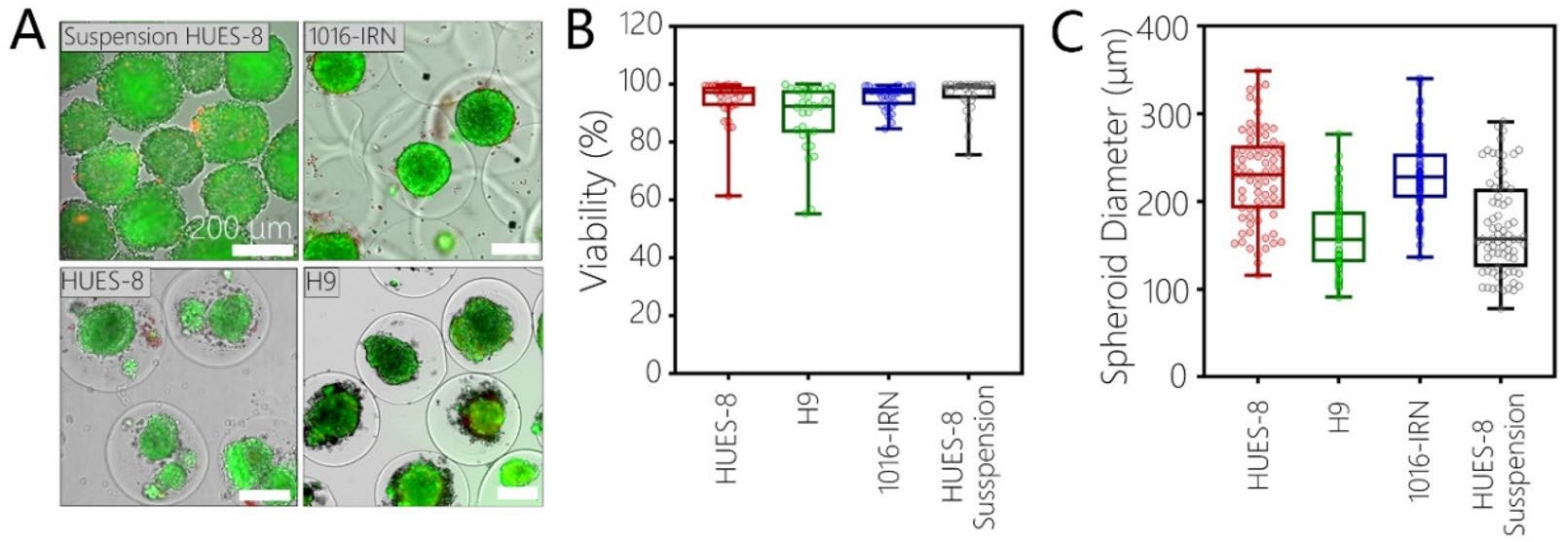
Encapsulation and spheroid formation of hPSC cell lines. (**A**) Images demonstrating encapsulation and spheroid formation for three hPSC lines. Live/Dead staining revealed that spheroids remained viable after encapsulation. Viability was similar for HUES-8 spheroids formed in standard suspension cultures and in microcapsules. Scale bars: 200 μm. (**B**) Viability quantified based on live/dead staining images of spheroids after 72 h of culture (n = 30). (**C**) Characterization of spheroid diameter for three encapsulated hPSC lines and for HUES-8 cells in suspension (n = 75).

Unlike standard spheroid formation in stirred cultures which happens as result of random encounters of cells floating in a large volume of media, our encapsulation strategy confines cells to a minute volume (~ 33 nL for a capsule with 400 μm diameter), thus increasing local cell concentration and forcing cellular interactions inside capsules. As highlighted by Fig. 2A, encapsulation resulted in spheroid formation for all three hPSC lines. Live/Dead staining revealed that viability of encapsulated stem cell spheroids was 94.4 ± 7.7% for HUES-8 cells, 88.7 ± 11.8% for H9 cells and 95.7 ± 4.1 for 1016 cells, compared to 96.5 ± 5.7% for HUES8 control spheroids (see Fig. 2A,B). Viability of bare and encapsulated HUES-8 spheroids was comparable, suggesting that encapsulation had no deleterious effects on stem cell survival. Furthermore, HUES8 cells had similar viability before and after encapsulation, further supporting the notion that the microfluidic encapsulation process was not damaging to cells (see Figure S2).

As shown in Fig. 2C the diameter for encapsulated spheroids was 228.3 ± 53.3 μm (CV-23.3%) for HUES8, 161 ± 40.2 μm for H9 (CV-25%), 230.1 ± 41.7 μm for 1016 (CV-18.1%) compared to 170.5 ± 54.1 (CV-31.7) for HUES8 spheroids without capsules.

The variability in the average spheroid diameter between the cell lines is likely due to the cell-line specific differences in cell-adhesive properties.

### Improving stem cell loading efficiency by integrating a microfluidic dissociation module in line with a flow focusing encapsulation device

While microfluidic encapsulation resulted in formation of highly viable stem cell spheroids, a significant fraction (~ 43%) of microcapsules did not have cells. Given the high input concentration of cells in the core flow stream (e.g. 30 or 60 × 10^6^ cells mL^−1^), most, if not all microcapsules were expected to contain cells/spheroids under ideal circumstances. However, under experimental conditions, cells were observed to clump in the syringe delivering core stream into the microfluidic encapsulation device. Changing orientation of the syringe (vertical vs. horizontal) and placing syringe on ice decreased but did not eliminate cell clumping. Because of clumping, cells were not uniformly distributed in the core stream leading to some capsules being empty and other capsules carrying an excess of cells.

**Figure 3:**
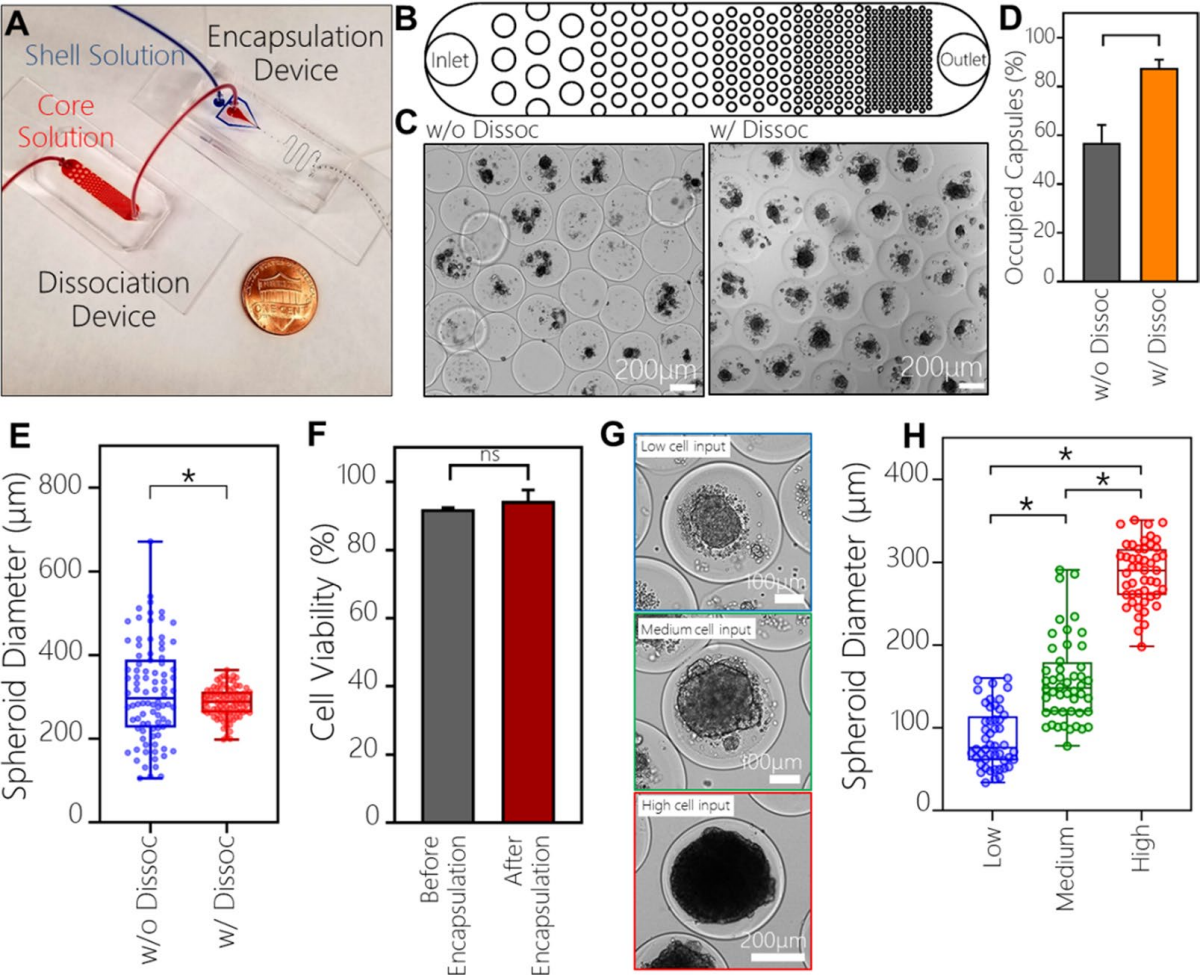
Integration of microfluidic dissociation and encapsulation modules. (**A**) Image of a microfluidic dissociation device interconnected with a flow focusing encapsulation device. Outlet from the dissociation device was connected to the core flow inlet of the encapsulation device. (**B**) Design of the dissociation device consisting of PDMS pillars with pitch ranging from 500 μm at the inlet to 50 μm at the outlet. (**C**) Microcapsules imaged immediately after fabrication. Note the number of blank capsules is lower when dissociation device is used prior to encapsulation. (**D**) Quantification of capsules occupied by cells (n = 100, *p* < 0.05). (**E**) Size distribution of spheroids after encapsulation with and without the dissociation device (n = 90, spheroids, *p* < 0.05). (**F**) HUES-8 viability is not affected by the dissociation/encapsulation process. (**G**) Images of encapsulated spheriods of HUES-8 cells created from varying starting cell concentrations. (**H**) Quantiation of spheroid diameters created from different cell inputs: 15 × 10^6^ cells mL^−1^ (low), 30 × 10^6^ cells mL^−1^ (medium) and 60 × 10^6^ cells mL^−1^ (high) (n = 48, *p* < 0.05). HUES-8 cells were encapsulated.

To address this challenge, we integrated a microfluidic dissociation/filtration device upstream of the microfluidic encapsulation device (see Fig. 3A and Figure S3). Passive filters containing arrays of posts have previously been incorporated into microfluidic devices to retain cell clumps, thus minimizing channel occlusion while cell seeding^38–40^ or as a way to mechanically digest tissues by using flow^41^. Using this concept, we created a dissociation device that contained an array of PDMS pillars that spanned the height of the microfluidic channel (50 μm) with pitch varying from 500 µm at the inlet to 50 µm at the outlet (Fig. 3B). COMSOL modeling revealed that at the flow rate of 3 μL min^−1^ shear stress levels in the device varied from 0.05 to 0.16 Pa for 500 μm and 50 μm pitch respectively (see Figure S3). The highest levels of shear generated in the filter/dissociation device were reported in the previous studies to be non-damaging to cells^4^.

Based on the flow rate in the dissociation device and the volume of connecting tubing, cells were expected to arrive in the encapsulation device 60 s after exiting the dissociation device—an amount of time insufficient for clumping to occur. Indeed, connecting the dissociation device in series with microfluidic encapsulation module allowed us to improve the loading efficiency from 57 to ~ 90%. The improved cell loading efficiency is highlighted by images in Fig. 3C and is quantified in Fig. 3D. The use of the dissociation device also allowed us to improve the size uniformity of encapsulated spheroids. The data in Fig. 3E show the encapsulated spheroids created using initial concentration of 60 × 10^6^ cells mL^−1^ were 310.8 ± 116.5 µm in diameter (CV = 37.5%) without dissociation compared to 286.9 ± 36.2 µm in diameter (CV = 12.6%) with the dissociation device. Importantly, viability of HUES-8 cells was unaffected by the dissociation/encapsulation process (see Fig. 3F).

Having improved uniformity of spheroid formation, we proceeded to demonstrate that spheroid size may be controlled by the inoculation density of stem cells. As shown in Fig. 3G,H, HUES-8 input concentrations of 15 × 10^6^ to 30 × 10^6^ to 60 × 10^6^ cells mL^−1^ resulted in spheroid diameters of 87.6 ± 35.6 µm, 157.2 ± 50.7 µm and 286.8 ± 36.7 µm. It is worth noting that the concentration of 15 × 10^6^ cells·mL^−1^ is not sufficient to form spheroids in stirred flasks without microcapsules. Given extensive evidence of spheroid size affecting differentiation fate of hPSCs^8,37,42,43^, our ability to control spheroid size and uniformity will have important implications for future efforts to differentiate encapsulated spheroids.

### Characterizing shear stress effects in a stirred bioreactor

**Figure 4:**
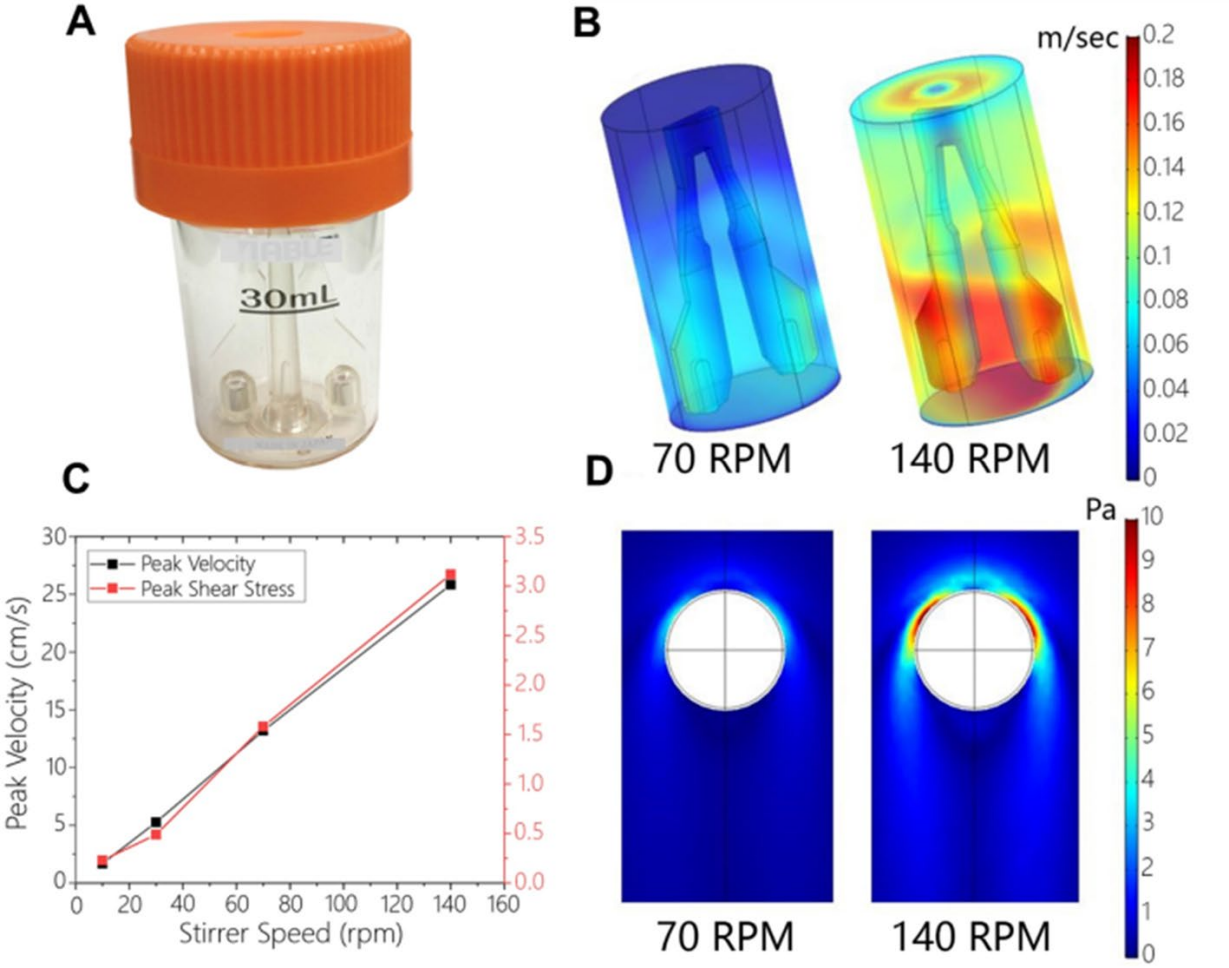
CFD modeling of shear stress and velocity profiles in the bioreactor. (**A**) Image of the bioreactor used to culture spheroids. (**B**) Velocity (m s^−1^) profiles within the bioreactor 3 s after initiation of stirrer speeds of 70 and 140 rpm. Impeller regions farthest away from the axis of rotation experienced the highest velocity magnitudes. (**C**) Peak velocity (cm s^−1^) and shear stress (Pa) in the bioreactor as a function of stirrer speed at t > 5 s (steady) after initiation of stirring showing linear dependency. (**D**) Slices of shear stress fields around the microcapsule (white circle) exposed to peak velocities of 70 and 140 rpm. Regions of high shear stress can be seen as dark red spots.

Stirred bioreactors utilize agitators or impellers to disperse spheroids throughout the reactor volume while efficiently delivering nutrients and oxygen. The speed of rotation or the shear stress generated by the impeller is therefore coupled to the rate of nutrient and oxygen delivery. As a result, suspension spheroid cultures strive to identify the “safe zone” for stirring where transport is adequate and shear stress is not damaging^25,44^. Previously, we empirically determined that HUES-8 cells stirred at the rate of 70 rpm formed ~ 200 µm diameter spheroids that remained undamaged and healthy during the multi-day culture protocol^37,45^. In the present study, we wanted to understand the shear stress and transport properties in the bioreactor operating at 70 rpm and to assess how these properties change in the event of increasing stirring speed. To address these questions, we set up computational fluid dynamics (CFD) model of the bioreactor (see Fig. 4A for image of the bioreactor) in COMSOL. Modelling of oxygen transport (Figure S5) revealed that oxygenation may be improved threefold by increasing the stirring speed from 70 to 140 rpm, with oxygen concentration reaching 3 μmol (or 1 mM) in the bioreactor at 140 rpm compared to 1 μmol at 70 rpm. Modulating oxygen levels during hPSC differentiation may be particularly useful given that cultures at early developmental stages have been shown to benefit from hypoxia while resultant adult cells (e.g. pancreatic β-cells or hepatocytes) have high demands for oxygen^46,47^.

Additional CFD modeling (see Fig. 4B) revealed magnitude of velocity within the reactor at stirrer speeds of 70 and 140 rpm, 3 s after stirring was initiated. An earlier time was chosen to show the development of mixing in the bioreactor as well as to show key differences between the stirrer speeds. In general, highest velocity magnitudes were seen on the impeller blades farthest from the rotating axis. This is expected as the tangential velocity at the tip of an impeller is proportional to radial distance from the axis of rotation. These results highlight that velocity profile inside the stirred bioreactor is complex/non-uniform. The peak velocity and shear stress dependence on the stirring speed at t > 5 s after initiation of stirring is described in Fig. 4C. It is worth noting that shear stress in the range of ~ 3 Pa has previously been reported damaging to cells cultured in suspension stirred bioreactors^18^. When modeling exposure of individual spheroids to peak bioreactor velocities, heterogeneous surface shear stress profiles were predicted (Fig. 4D). Subjecting spheroids to the peak velocity under a stirrer speed of 140 rpm produced relatively high local shear stresses (~ 10 Pa) on spheroid surfaces. We therefore expected to observe shear stress effects in suspension cultures at higher speed of rotation.

**Figure 5:**
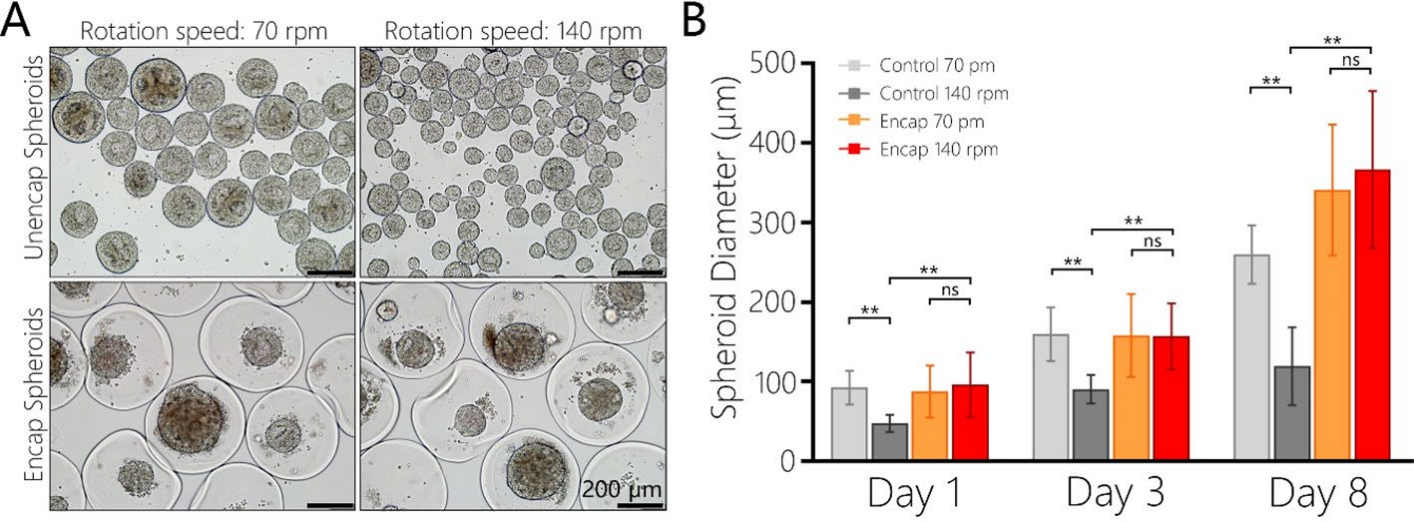
Assessing the effects of the speed of stirring on the size of spheroids. (**A**) Representative brightfield images of stem cell spheroids after 3 days of suspension cultures at 70 rpm and 140 rpm. (**B**) Analysis of spheroid diameter for encapsulated and control spheroids at 70 rpm and 140 rpm (n = 20, **p < 0.01).

To experimentally confirm the effects of shear stress in our bioreactor, bare and encapsulated HUES-8 spheroids were cultured for 8 days at either 70 rpm or 140 rpm. Images in Fig. 5A highlight the differences between experimental groups: (1) bare spheroids stirred at 70 rpm were larger than those spheroids cultured at 140 rpm, (2) encapsulated spheroids were of similar diameter for both stirring conditions. Quantification of spheroid sizes (see Fig. 5B) revealed that no significant differences existed between diameters of encapsulated spheroids cultured for 3 days at 70 and 140 rpm (158.2 ± 11.3 µm vs. 156.8 ± 9.1 µm). By contrast, the diameters for bare spheroids were 159.8 ± 7.4 and 90.4 ± 2.8 μm for 70 and 140 rpm respectively. This result points to the effects of shear on bare spheroids at higher speeds of rotation and highlights the protection from the shear stress provided by the hydrogel microcapsule. During this experiment, we did not observe appreciable damage (e.g. rapture) of microcapsules at 140 rpm and can infer that the microcapsules are mechanically strong enough to withstand the shear stress in excess of 3 Pa.

### Assessing pluripotency expression and differentiation potential of hPSC spheroids

In the preceding sections of the paper we demonstrated that microcapsules may be leveraged to ensure spheroid formation and improve spheroid size uniformity as well as to protect cells against shear stress. In this section, we wanted to evaluate pluripotency state and differentiation of encapsulated hPSCs in comparison with unencapsulated spheroids. We considered two types of hPSC spheroid controls without capsules for these experiments. Control 1—hPSCs placed into syringe containing core components (densifier, non-crosslinkable high MW PEG) for the duration of the encapsulation run (~ 1 h) and then formed into spheroids in a bioreactor. Control 2—hPSCs formed into spheroids in a bioreactor without prior exposure to chemicals present during microencapsulation. Comparison of pluripotency and endodermal gene expression revealed no significant differences between the two types of control conditions (see Figure S6). Therefore, we used spheroids directly formed in the bioreactor as controls for encapsulated spheroids. We should also note that all pluripotency and differentiation experiments described below were carried out with HUES-8 cells.

**Figure 6:**
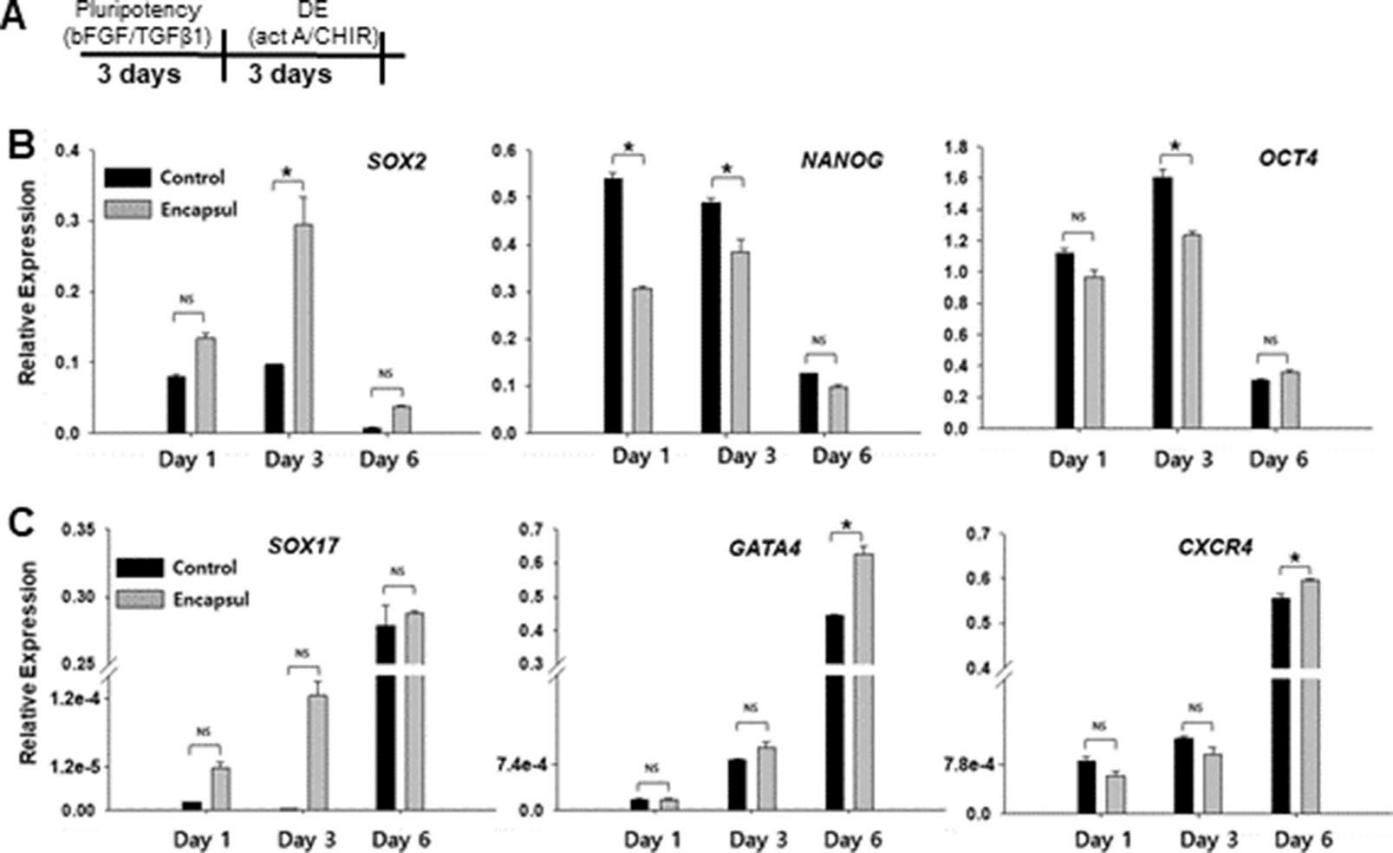
Evaluation of plurpotency maintenance and endodermal differentiation of hPSC spheroids. (**A**) Workflow of the pluripotency maintenance and endodermal differentiation experiment. (**B**) RT-PCR analysis of pluripotency genes OCT4, SOX2, NANOG. For statistical analysis n = 4, *p* < 0.05. (**C**) RT-PCR analysis of endodermal genes SOX17, GATA4, CXCR4. (n > 4, *p* < 0.05).

Having identified appropriate control conditions, we proceeded to assess pluripotency state and differentiation potential of encapsulated spheroids. In the first set of experiments (see Fig. 6A), hPSC spheroids were cultured in pluripotency maintenance media (mTeSR) for 3 days and then transferred to endodermal differentiation media containing activin A for another 3 days. Pluripotency and endodermal gene expression was monitored by RT-PCR over the course of this experiment. Characterization of gene expression revealed an expected trend for both encapsulated and control spheroids—expression of pluripotency genes (*OCT4, SOX2, NANOG*) was high with cells in pluripotency (mTeSR) media at days 1 and 3 but then decreased after spheroids were transferred into endodermal differentiation media during subsequent 3 days (Fig. 6B,C). Conversely, expression of endodermal genes (*SOX17*, *GATA4*, *CXCR4*) was low in pluripotency media at days 1 and 3 but high at day 6 after endodermal differentiation. While statistically significant differences in pluripotency gene expression were observed between control and encapsulated spheroids, no trend pointing to one condition being better than the other could be discerned (Fig. 6B). *SOX2* expression was higher at day 3 in encapsulated spheroids whereas *OCT4* and *NANOG* exhibited higher expression at day 3 in control spheroids. Importantly, induction of endoderm drove endodermal gene expression of encapsulated spheroids to the level similar or better than control spheroids (see Fig. 6C). This result supports the notion that microencapsulation does not adversely affect pluripotency state and endodermal differentiation of hPSC spheroids. Further evidence for this is provided by β-cell differentiation experiments described below.

**Figure 7:**
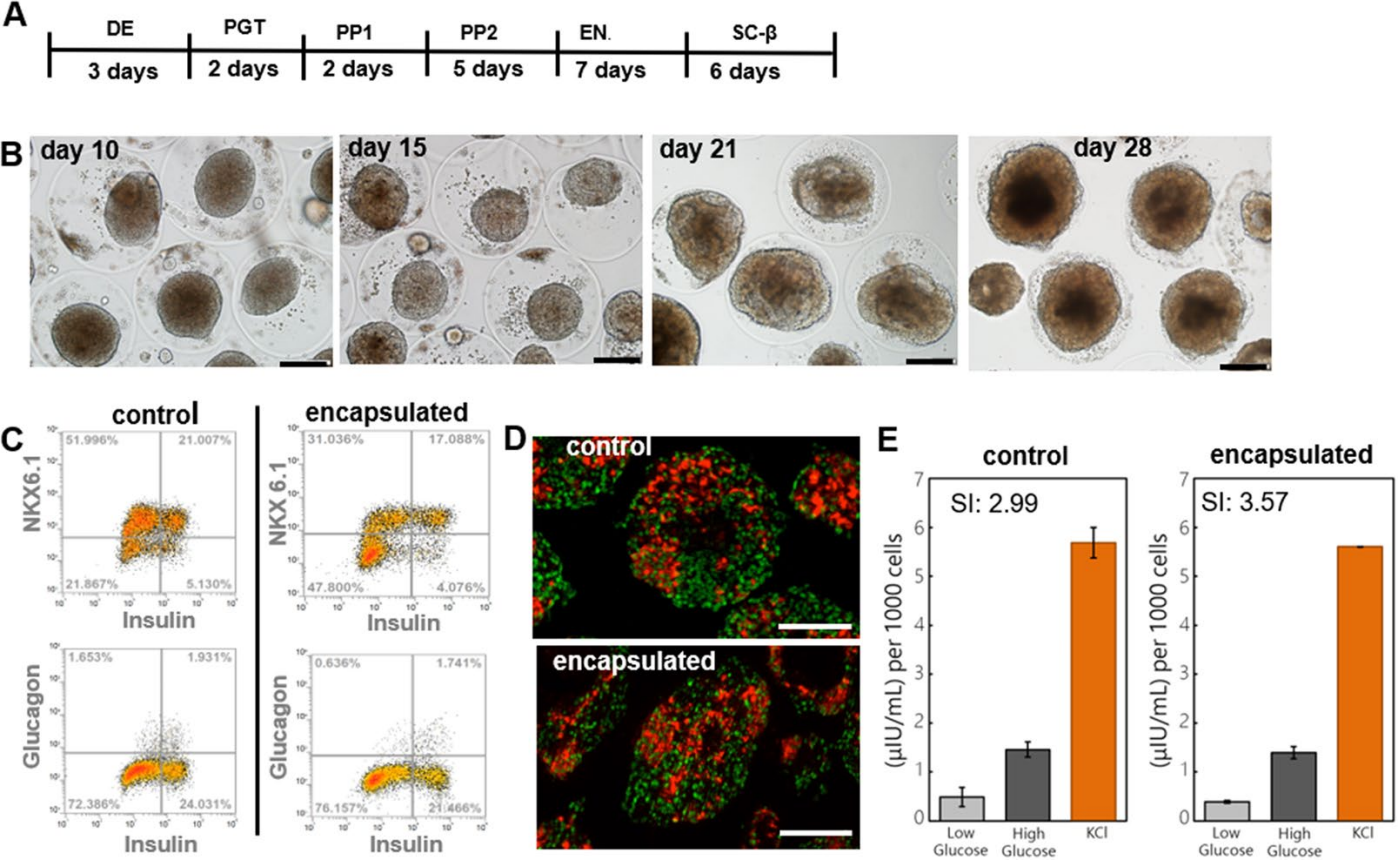
Differentiation of hPSC spheroids into pancreatic β-cells. (**A**) A multi-stage differentiation protocol with some of the key inductive signals at each stage. HUES-8 spheroids were differentiated in a stirred bioreactor according to this protocol. (**B**) Images of encapsulated spheroids at different time points during differentiation. Scale bar—150 μm. (**C**) Flow cytometry analysis after completion of the 4 week differentiation protocol. Cells from dissociated spheroids were labeled for endocrine pancreatic marker NKX6.1, β-cell-specific marker insulin and α-cell marker glucagon. β-cells were characterized as NKX^+^/insulin^+^/glucagon^−^. (**D**) Immunofluorescence staining of intact spheroids for NKX6.1 (green fluorescence) and insulin (red fluorescence). Scale bar—200 μm. (**E**) Functional analysis of β-cell spheroids using glucose stimulation insulin secretion assay. Stimulation index (SI) is the ratio of insulin produced by β-cells after high and low glucose challenges.

In the second set of experiments, encapsulated hPSC spheroids were directed toward pancreatic β-cell lineage using a previously multi-stage differentiation protocol described in Fig. 7A and previously published reports^12,37^. In this protocol, stem cell spheroids cultured in a stirred bioreactor were guided by sequential and timed administration of inductive signals through a series of developmental stages reminiscent of endocrine pancreatic development. These stages were: definitive endoderm (DE), primitive gut tube (PGT) formation, pancreatic progenitor (PP) stages 1 and 2, endocrine progenitor stage (EN) and a stem cell-derived β-cell stage (SC-β). At the end of the protocol (after 26 days), β-cell markers and function were characterized for control and encapsulated spheroids.

Figure 7B shows representative images of encapsulated spheroids at different times during differentiation. It may be appreciated from these images that over the course of 4 weeks, spheroids undergo significant changes in size/morphology and that capsules accommodate such changes and remain structurally intact for at least three weeks. As seen from Fig. 7B capsules begin experiencing structural breakdown after 4 weeks of culture. This breakdown is likely caused by continued stretching of the hydrogel shell by the growing spheroids and may be mitigated in the future by increasing capsule diameter or decreasing hPSC inoculation density.

The presence of the hydrogel shell is desirable for diabetes-related applications where β-cells are typically transplanted ectopically, function in an endocrine manner and benefit from immunoprotective coatings. However, other applications involving tissue regeneration or repair may call for degradable capsules. Several strategies for designing degradable hydrogels have been reported in the literature and may be pursued in the future to degrade spheroid-carrying microcapsules^48–52^.

Efficiency or yield of β-cell differentiation was assessed by flow cytometry and immunofluorescence staining. Flow cytometry revealed that in-capsule differentiation resulted in 17% of cells expressing pancreatic β-cell markers insulin and NKX6.1 and these cells were negative for glucagon—a marker of pancreatic α-cells. Importantly, populations of NKX6.1^+^ insulin^+^ and glucagon^−^ insulin^+^ cells were similar for encapsulated and control spheroids, 17% and 21% respectively (Fig. 7C). Immunofluorescent staining and imaging of intact spheroids confirmed that a large fraction of cells within a spheroid stained positive for β-cell markers, NKX6.1 and insulin (Fig. 7D).

To further assess functionality of β-cells, we carried out glucose stimulation insulin secretion (GSIS) analysis where insulin secretory capacity of the cells is evaluated by sequential glucose challenges followed by depolarization with KCl^37^. Figure 7E summarizes results of GSIS analysis for encapsulated and control spheroids. As seen from these data, both types of spheroids secreted insulin in response to glucose challenges with secretion index of 2.99 and 3.57 for control and encapsulated spheroids respectively. This result provides additional evidence in support of encapsulation process being compatible with production of highly functional cells exhibiting markers of pancreatic β-cells.

## Conclusions

In this study we sought to address the need for an hPSC encapsulation technology that would result in formation of viable stem cell spheroids of uniform and tunable size. We also assessed the utility of microcapsules for shielding cell spheroids against shear stress in the stirred bioreactor. To achieve the goals of this study, we utilized a flow focusing microfluidic device to fabricate microcapsules with a hydrogel shell and an aqueous core. These microcapsules were shown to be conducive to rapid aggregation of hPSCs into spheroids. Successful encapsulation and formation of high viability spheroids was demonstrated for three different hPSC lines.

The challenge of inefficient cell loading due to clumping, experienced by us during initial phase of this study, was resolved by integrating a dissociation device in line with and upstream of the microencapsulation device. By using this integrated encapsulation platform, the frequency of spheroid-containing capsules was improved from 57 to 90% while the distribution of spheroid size narrowed from 310.8 ± 116.5 to 286.9 ± 36.2 µm in diameter for high density loading.

In addition, we carried out CFD modeling of transport in the stirred bioreactor used for cultivation of spheroids. This modeling predicted that the increase in stirring speed may result in shear stress levels that are damaging to stem cell spheroids and potentially improve the oxygenation of the stirred bioreactor. We then demonstrated experimentally that such damage is observed in bare spheroids which decrease in size at higher stirring speeds but is not observed in encapsulated spheroids. Our experimental results suggest that hydrogel shell of the microcapsules is shielding spheroids from the shear stress reaching 3 Pa. The use of hydrogel microcapsules offers the benefit of regulating/improving transport properties without damaging the cells, thus allowing one to uncouple transport from shear stress in a stirred bioreactor.

Importantly, encapsulation did not affect pluripotency state and differential potential of hPSC spheroids. The latter point was highlighted by successful differentiation of encapsulated hPSC spheroids into cells expressing markers and function of pancreatic β-cells.

In the future, the microcapsules may be further improved for ease of hydrogel degradation/spheroid retrieval as well as for controlled loading and release of inductive signals. The microcapsules may also be used as vehicles for transplantation of stem-cell derived adult cells into immune competent animal models.

## Experimental section

### Fabrication of microencapsulation devices

Microencapsulation flow-focusing devices were fabricated according to a previously described protocol^35^. The polydimethylsiloxane (PDMS) microfluidic device consists of two mirrored triple-height PDMS microfluidic devices bonded together to achieve a non-planar (3D) flow-focusing device. Briefly, the microfluidic device was designed in AutoCAD (version 2019, Autodesk Inc.). Based on device design, two master molds, original design (top) and mirror of it (bottom), were fabricated by multi-step photolithography using SU-8 2050 photoresist (MicroChem, Westborough, MA) to achieve three different heights: (1) 50 µm for aqueous core solution, (2) 100 µm for aqueous shell solution and (3) 150 µm for oil phase. Two 4-inch silicon wafers (University Wafer, USA) were spin coated with a 50 µm layer of photoresist, soft baked (5 min at 65 °C, 10 min at 95 °C) and exposed to UV light through a photomask with the desired structures using a mask aligner (UV-KUB 3, Kloé, France). A post exposure bake was performed to both molds for 3 min at 65 °C and 10 min at 95 °C. Features were developed by direct submersion in SU-8 developer (MicroChem, Westborough, MA) until unexposed photoresist was completely removed. Afterwards, a 100 µm layer of photoresist was spin coated on top of the 50 µm layer and structures were exposed with a similar protocol to the one just described, changing soft bake to 5 min at 65 °C and 20 min at 95 °C. The third photoresist layer of 150 µm was spin coted on top of the previous two and a similar protocol was followed to expose structures, changing soft bake (5 min at 65 °C, 30 min at 95 °C) and post exposure bake (5 min at 65 °C, 15 min at 95 °C) times. Finally, both molds were placed on a hot plate at 160 °C for 10 min as hard bake, followed by exposure to chlorotrimethylsilane for 30 min on a closed chamber to avoid PDMS adhesion to master molds.

After mold fabrication, PDMS (Sylgard 184 silicone elastomer kit, Dow Corning) replicas were fabricated by soft lithography^53^. For this, 55 g of PDMS mixture with a ratio of 10 to 1 of base and curing agent was poured into each master mold, degassed for 10 min on a vacuum desiccator and baked for 60 min at 80 °C on a convection oven. Cured “top” and “bottom” PDMS pieces were cut, inlets and outlets punched out and treated with oxygen plasma for 45 s (G-500 plasma cleaning system, Yield Engineering Systems, Livermore, CA). PDMS pieces were manually aligned under stereoscope (Zeiss Stemi 508, Germany) using deionized water as a lubricating layer. Aligned PDMS devices were then placed in an oven at 80 °C overnight to remove water layer.

Final PDMS microencapsulation devices contained channel with three heights: (1) 100 µm for aqueous core channel, (2) 200 µm for aqueous shell channels, and (3) 300 µm for oil and capsule collection channels. Microencapsulation devices were placed on a glass slide for handling and channels are treated with Aquapel solution previous to use to render the surfaces hydrophobic.

### Fabrication of dissociation/filtering devices

To improve cell-seeding into capsules, a microfluidic dissociation device was fabricated. The microfluidic filter consists of a single layer device with a height of 50 µm, fabricated with the same protocol as the microencapsulation device. The device comprises a simple rectangular chamber with an inlet and an outlet, as shown in Figure S1. The chamber contained an array of posts with larger diameter and larger separation towards the inlet, becoming smaller in diameter and closer to each other closer to the outlet of the device. This array of posts allowed trapping and dissociation of larger cell aggregates while letting cell clumps < 50 μm enter the encapsulation system.

### Microfluidic encapsulation of hPSCs

We followed a previously described protocol^35^ for encapsulation of hPSCs. Briefly, the core and shell fluids were a 50:50 ratio of 16% w/v PEG (35 kDa) and 34% Optiprep densifier (Sigma-Aldrich, St. Louis, MO) dissolved in Krebs–Ringer bicarbonate (KRB) buffer and 10% (range 7–12%) w/v 4-arm maleimide functionalized poly(ethylene glycol) (PEG 10 k) and 15 mM triethanolamine (TEA) (range 10–20%) dissolved in DI water, respectively. The shielding oil phase at the second flow-focusing cross-section consisted of mineral oil and 1.5% (w/v) of surfactant Span80 (Sigma-Aldrich, St. Louis, MO). To make the crosslinking oil for flowing in the crosslinking channel a solution of mineral oil with 3% (w/v) Span-80 mixed with dithiothreitol (DTT) (Ratio of 1:15 emulsion of 25 mg mL^−1^ DTT) dissolved in DI water was prepared by sonication for 45–60 min in an ultrasonic bath at 20 °C. All the solutions are injected into the inlets of the microfluidic device through Tygon Microbore Tubing (Dimensions: 0.020″ ID × 0.060″ OD). Flow rates of the streams were regulated via syringe pumps (Harvard Apparatus, Holliston, MA) at the following rates: core: 3–5 μL min^−1^, shell: 3–5 μL min^−1^, shielding oil: 30–50 μL min^−1^, and crosslinking oil: 40–60 μL min^−1^.

Cell suspensions were loaded into the core solutions at three different concentrations: low (15–20 × 10^6^ cells mL^−1^), medium (30–35 × 10^6^ cells mL^−1^) and high (50–60 × 10^6^ cells mL^−1^). At the experimental flow rates, the frequency of our core/shell microencapsulation is ~ 5–6 capsules per second with each capsule containing ~ 300 cells at medium cell concentration (see below). For medium cell concentration, ~ 7.6 × 10^6^ cells could be encapsulated in 1 h of operating the microfluidic system.

HPSCs tended to aggregate in the syringe before reaching the encapsulation system causing a significant fraction (~ 43%) of capsules to be empty of cells. The aggregation was decreased, but not eliminated, by keeping cells at 4 °C. Dissociation/filter microfluidic module was used to address this challenged. The dissociation and microencapsulation units were integrated using 5–7 cm Tygon Microbore Tubing (Dimensions: 0.020″ ID × 0.060″ OD), with one end of the tube placed into the outlet of the dissociation device and the other end inserted in the “core” flow input of the encapsulation device. Therefore, shell and oil solutions were directly injected into the encapsulation device whereas core solution with stem cells was routed through the dissociation device first. Core solutions loaded with cell suspensions were perfused using a syringe pump (Harvard Apparatus, USA) at a flow rate of 4 µL min^−1^.

### Cultivation of hPSC spheroids

HUES-8 and H9 cells (both hESC lines) as well as 1016 (hiPSC line) were cultured as spheroids in 30 mL spinner flasks (ABLE Biott, Japan). Suspension cultures were established by seeding 15 million cells (5 × 10^5^ cells mL^−1^) in mTeSR media (STEMCELL Technologies, Vancouver, Canada) with 10 µM Y27632 (STEMCELL Technologies, Vancouver, Canada). The spinner flasks were placed on a stir plate at the speed of 70 rpm inside the humidified incubator at 37 °C, and 5% CO_2_. Media was changed at 48 h to mTeSR without Y27632. Cells were passaged every 72 h by dispersing to single cells using Accutase and resuspended in fresh mTeSR with Y27632.

When assessing effects of shear stress, stem cells spheroids were kept in mTeSR media for 3 days. For additional 5 days, spheroids were maintained in the MCDB131-based media with additives described in detail in our previous work as S1 media^37^. The latter media was used to minimize proliferation of spheroids.

### Assessing cell viability

A live/dead cell imaging kit (488/570, Thermo Fisher Scientific) was used to assess cell viability of microcapsules with stem cell spheroids. Staining solution was prepared on a 15 mL tube by mixing 10 mL of media, 20 µL of ethidium homodimer and 5 µL of Calcein-AM (Live/Dead Viability/Cytotoxicity Kit, Thermo Fisher Scientific). Microcapsules were placed in the media and incubated for 30 min at room temperature. Staining solution was washed using 1X PBS. The bright-field and fluorescence images were taken using an inverted microscope (IX-83, Olympus).

### Bioreactor modeling and fluid dynamics simulations

Two types of simulations were carried out to determine the effect of mixing on microcapsules. In the first simulation, a model of the bioreactor was constructed. The bioreactor used in the experiments has a magnetically driven stirrer (ABLE Biott, Japan) and has a nominal volume of 30 mL. The reactor and the stirrer were 3D-scanned and a geometric model was constructed in COMSOL (COMSOL, MA). Computational fluid dynamics simulations were carried out using the CFD module in COMSOL under isothermal laminar flow conditions using material properties of water (incompressible Newtonian fluid). The rotational speed was varied: 10, 30, 70 and 140 rpm. Unsteady simulations were carried out with the rotational speed at t > 0 set to the prescribed value for > 5 s. Mesh optimization was carried out using various physics-based sizing options (coarse, normal, fine and extra-fine) within the software. A mesh size of normal was deemed to be sufficient; no appreciable changes in results were observed by further reducing the mesh size. Peak velocity in the bioreactor for each rotational speed was determined. To determine the shear stress profiles around the microcapsules, in the second simulation, a model of microcapsule was constructed and exposed to the peak velocity in a volume relatively large (10×) to its size. The above twostep process allowed us to separately obtain reactor and microcapsule-specific information.

### Analysis of pluripotency

Pluripotency of encapsulated and bare spheroids was assessed by RT-PCR. Both types of spheroids were cultured for up to 3 days in stirred bioreactor in mTeSR media and then collected for analysis. The microcapsules were first broken by mechanical agitation (pipetting up and down). Liberated cells were then lysed and total RNA was isolated using mRNA extraction kit (Roche). The RNA abundance and quality were assessed by NanoDrop UV–Vis spectrophotometer (Thermo Fisher Scientific).

The total mRNA was then converted to cDNA using a Transcriptor First Strand cDNA synthesis kit (Roche) and analyzed using a QuantStudio 3 Real-Time PCR System (Thermo Fisher Scientific) for the expression of *OCT4*, *NANOG* and *SOX2* genes. Predesigned TaqMan probes for pluripotency genes were purchased from Thermo Fisher. Pluripotency gene expression was quantified relative to GAPDH housekeeping gene using the ∆∆Ct method.

### Endodermal and β-cell differentiation of hPSC spheroids

HUES-8 cells were used for all differentiation experiments described below. The differentiation began after 3 days of stem cell spheroid formation and expansion in mTeSR media inside a stirred bioreactor. Approximately 60 × 10^6^ cells were present in the bioreactor during a differentiation run. We followed previously described differentiation protocols^37,54^. Basal media types, numbered S1, S2, S3, and BE5; and were supplemented with inductive signals as described below.

**S1 media**, was comprised of 500 mL MCDB 131 supplemented with 0.22 g glucose, 1.23 g sodium bicarbonate, 10 g fatty acid free bovine serum albumin (FAF-BSA, Proliant Biologicals), 10 μL ITS-X, 5 mL GlutaMAX, 22 mg ascorbic acid, and 5 mL penicillin/streptomycin (P/S) solution. **S2 media:** 500 mL MCDB 131 supplemented with 0.22 g glucose, 0.615 g sodium bicarbonate, 10 g FAF-BSA, 10 μL ITS-X, 5 mL GlutaMAX, 22 mg ascorbic acid, and 5 mL P/S. **S3 media:** 500 mL MCDB 131 supplemented with 0.22 g glucose, 0.615 g sodium bicarbonate, 10 g FAF-BSA, 2.5 mL ITS-X, 5 mL GlutaMAX, 22 mg ascorbic acid, and 5 mL P/S. **BE5 media:** 500 mL MCDB 131 supplemented with 1.8 g glucose, 0.877 g sodium bicarbonate, 10 g FAF-BSA, 2.5 mL ITS-X, 5 mL GlutaMAX, 22 mg ascorbic acid, 5 mL P/S, and 2000 units heparin (MilliporeSigma).

Directed differentiation of pluripotent stem cells to SC-β cells was performed by changing media within the spinner flask and supplementation with small molecules and growth factors specific to the differentiation stage. Media changes are as follows: Day 1: S1 media + 100 ng/mL Activin A + 3 mM CHIR99021; Day 2: S1 media + 100 ng/mL Activin A; Day 4: S2 media + 50 ng/mL KGF; Day 6: S3 media + 50 ng/mL KGF + 250 nM Sant-1 + 500 nM PDBu + 200 nM LDN 193189 + 2 µM RA + 10 µM Y27632; Day 7: S3 media + 50 ng/mL KGF + 250 nM Sant-1 + 500 nM PDBu + 2 µM RA + 10 µM Y27632; Days 8, 10, 12: S3 media + 50 ng/mL KGF + 250 nM Sant-1 + 100 nM RA + 10 µM Y27632 + 5 ng/mL Activin A; Days 13 + 15: BE5 media + 250 nM Sant-1 + 20 ng/mL betacellulin + 1 µM XXI + 10 µM ALK5i + 1 µM T3 + 100 nM RA; Days 17 + 19: 20 ng/mL betacellulin + 1 µM XXI + 10 µM ALK5i + 1 µM T3 + 25 nM RA; Days 20–26: S3 media only. KGF (cat # 100-19) was purchased from Peprotech, all other factors were purchased from R&D Systems with the following catalog numbers: Activin A (338-AC), CHIR (4423), SANT-1 (1974), PDBu (4153), RA, Retinoic Acid (0695), LDN, LDN193189 (6053), Y27632 (1254), Betacellulin (261-CE), ALK5i (3742), T3, L-3,3',5-Triiodothyronine (5552), XXI, γ-secretase inhibitor XXI (6476).

Endodermal phenotype was characterized after 3 days of differentiation by expression of *SOX17*, *GATA4* and *CXCR4* genes by RT-PCR. Predesigned TaqMan probes for these genes were purchased from Thermo Fisher. Gene expression was normalized by the GAPDH housekeeping gene using the ∆∆Ct method.

### Flow cytometry

After completion of the differentiation protocol, microcapsules were pipetted up and down to break the hydrogel shell and release spheroids. The spheroids were then dispersed into single-cells by incubation in TrypLE at 37 °C 15 min. Cell were fixed in 4% PFA for 20 min and stored at 4 °C until further use. Prior to staining, cells were incubated in the blocking solution (1x PBS, 0.1% Triton X-100, 5% donkey serum) at room temperature for 40 min and then washed in 1x PBS with 0.1% Triton X-100T. Cells were then incubated with primary antibodies in the blocking solution (see above) for 1 h at room temperature, washed twice with 1x PBS, 0.1% Triton X-100 and incubated with secondary antibodies in the block solution for 1 h at RT. The following primary antibodies were used for flow cytometry: rat anti-human antibody for insulin (DSHB, cat# GN-ID4), goat anti-human antibody for glucagon (Sigma, cat# G2654) and mouse anti-human antibody for NKX 6.1 (DSHB, cat#F55A12). Secondary antibodies used for flow cytometry were donkey-anti-rat-Alexa Fluor 488 (1:1000) and donkey-anti-mouse-Alexa Fluor 647. After labeling, cells were washed three times, resuspended in 1x PBS with 0.01% Tween 20 at a concentration of 1 × 10^6^ cells/mL and analyzed using Attune low cytometer.

### Immunostaining

Encapsulated and unencapsulated spheroids were placed into 4% PFA for 20 min for fixation and were then washed in 1x PBS. Spheroids were then embedded in Histogel, transferred into paraffin and sectioned into 5 μm slices using microtome. Sections were stained for insulin and NKX 6.1 using the antibodies and protocols described above and were mounted in Fluoromount-G with DAPI. Clusters were imaged using an inverted fluorescence microscope (IX-83, Olympus).

### Glucose stimulation insulin section experiments

Detailed protocols for glucose stimulation insulin secretion experiments are described elsewhere^37^. Briefly, approximately 10^6^ cells were placed into a transwell and allowed to equilibrate in low KRB (Krebs–Ringer Buffer) for 1 h. Following the initial equilibration period, transwells were transferred sequentially to KRB buffer containing low glucose (3.3 mM) high glucose (16.7 mM) and KCl (30 mM KCl). Following incubation, aliquots were taken from each well and analyzed for insulin using ELISA (ALPCO- Human Ultrasensitive Insulin).

### Statistical analysis

Data are represented as mean ± SD and mean ± SEM. Statistical significance between experimental groups was assessed using a two-tailed student’s t-tests and *p* values < 0.05 were considered statistically significant.

## Supplementary Information

Supplementary Informations.
