# Supplementary material for: Core–shell hydrogel microcapsules enable formation of human pluripotent stem cell spheroids and their cultivation in a stirred bioreactor
Source: Sci Rep. 2021 Mar 30;11:7177. doi: 10.1038/s41598-021-85786-2 (PMC8010084; doi:10.1038/s41598-021-85786-2)
Supplement: Supplementary file 1 — Supplementary Informations. [file 41598_2021_85786_MOESM1_ESM.docx]

Supporting Information

Core-Shell Hydrogel Microcapsules Enable Formation of Human Pluripotent Stem Cell Spheroids and Their Cultivation in a Stirred Bioreactor

Pouria Fattahi^1,†^, Ali Rahimian^1,†^, Michael Q. Slama^1^, Kihak Gwon^1^, Alan M. Gonzalez-Suarez^1^, Jadon Wolf^2^, Harihara Baskaran^2^, Caden D. Duffy^1^, Gulnaz Stybayeva^1^, Quinn P. Peterson^1^, and Alexander Revzin^1,^*

^1^Department of Physiology and Biomedical Engineering, Mayo Clinic, Rochester, MN, 55902, USA

^2^Department of Chemical and Biomolecular Engineering, Case Western Reserve University, Cleveland, OH 44106, USA

***** E-mail: Revzin.Alexander@mayo.edu

^†^ These authors contributed equally to this work


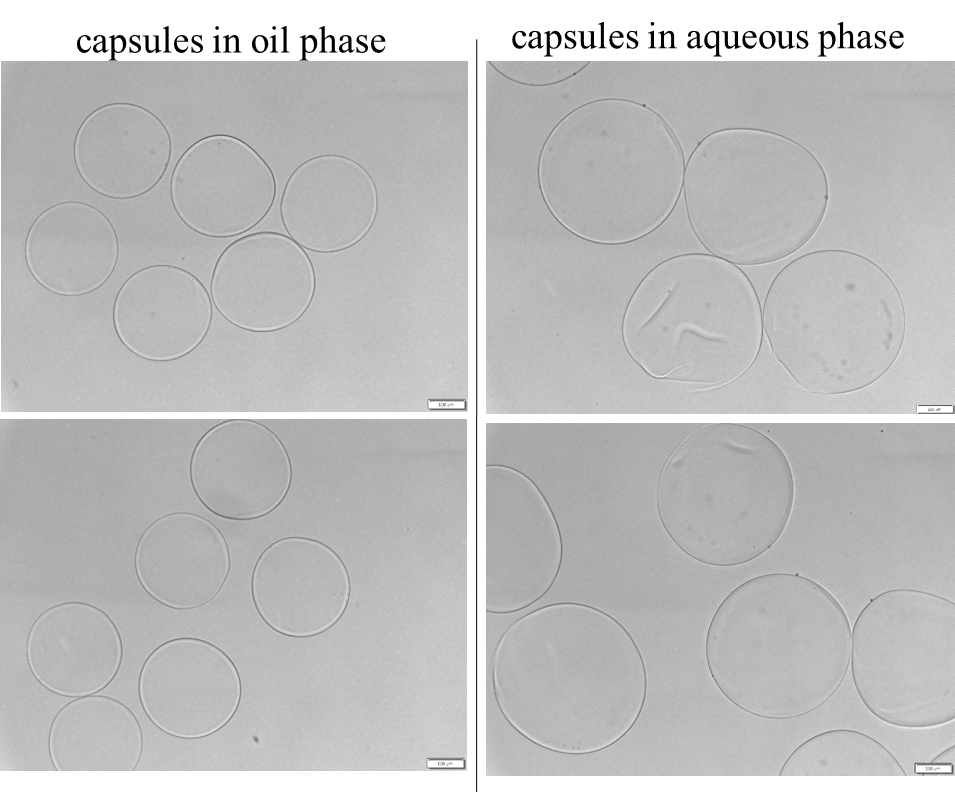


**B**

**A**

**Figure S1. Swelling of microcapsules in aqueous phase. (A)** Two representative images of hydrogel microcapsules in the oil phase, immediately after fabrication. **(B)** Two representative images of microcapsules after 2h in the aqueous phase. Images in (A) and (B) are collected at 10x magnification, scale bar – 150 μm.


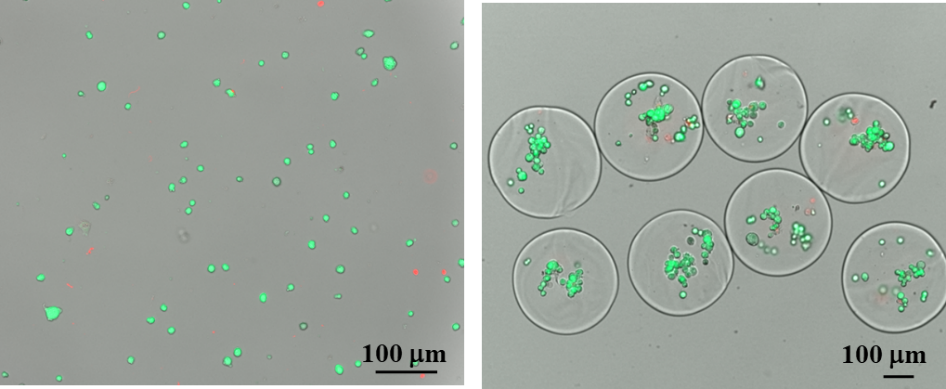


**Figure S2**. **Live/dead staining of HUES8 cells before and after encapsulation.**

Viability was determined to be 94.5 ± 1.4% before (left) and 94.3 ± 3.1% (right) after encapsulation. Analysis based on ~85 cells per image, 3 images per condition. No statistical significance between conditions for p<0.05.


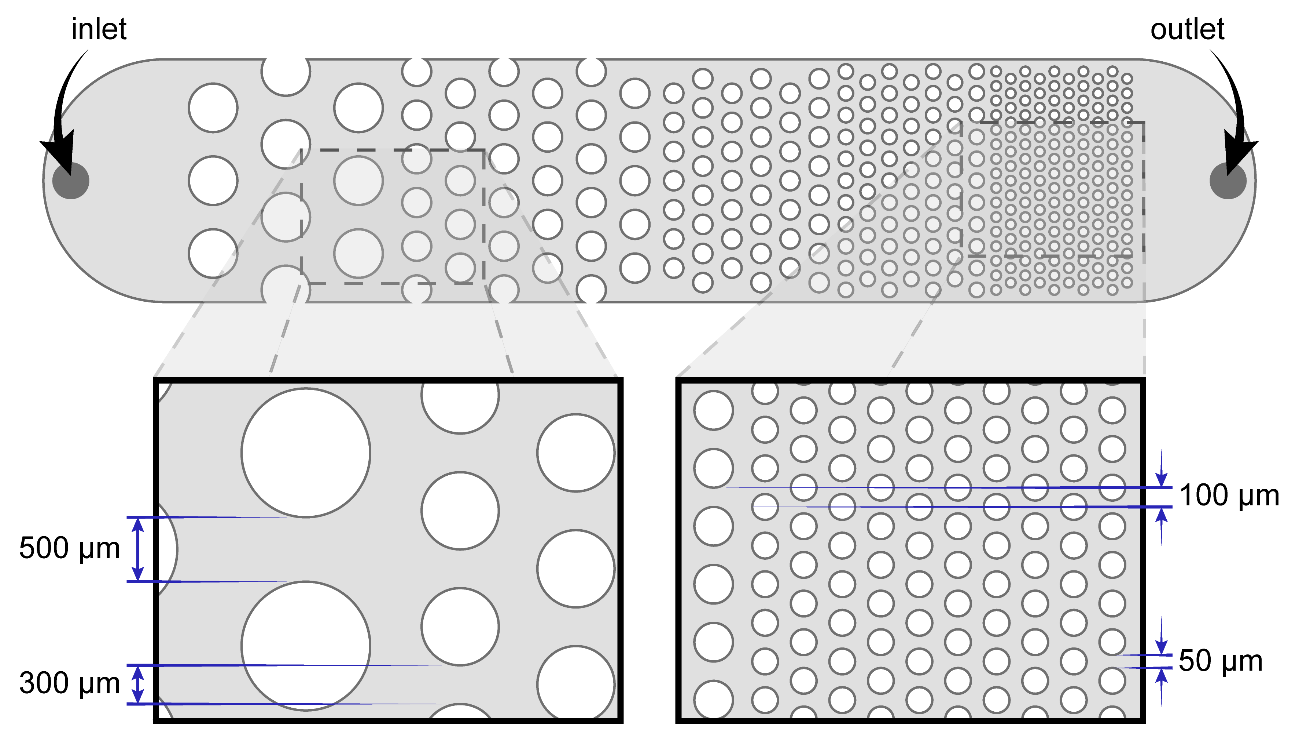


**Figure S3. Microfluidic dissociation device.** Design of the microfluidic dissociation device used to avoid large cell aggregates entering microencapsulation device. The device comprises an array of posts distributed in a long rectangular chamber with rounded ends. Post diameter and separation is larger (500 µm) closer to the inlet, and decreases (50 µm) closer to the outlet. Larger cell aggregates are retained and brokend down in the device, while cell aggregares with diameter <50 µm flow through the dissociation device and into the encapsulation device.


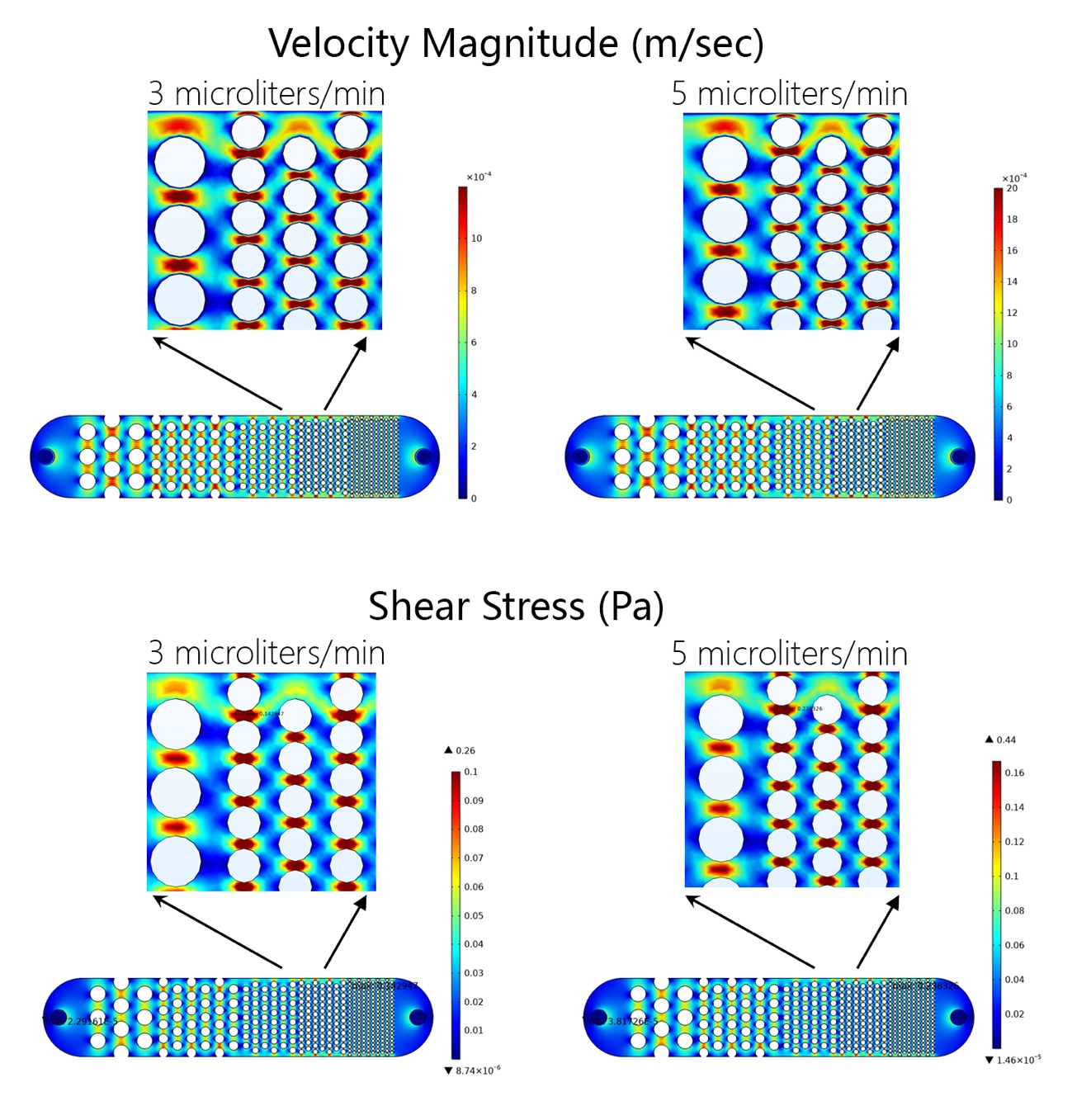


**Figure S4. Microfluidic dissociation device.** Velocity (m.sec^-1^) profiles and shear stress (Pa) profiles in the dissociation device at experimental flow rate.


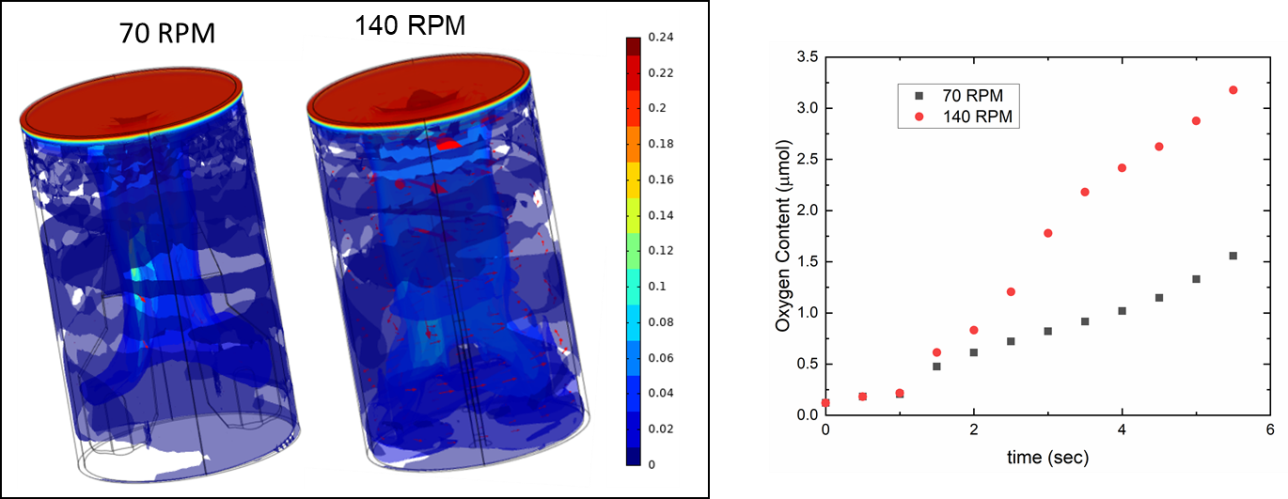


**B**

**A**

**Figure S5. CFD modeling of oxygen transport in a stirred bioreactor. (A)** Oxygen concentration (mol/m^3^) in the Reactor at 5 seconds after initiating mixing. Arrows represent oxygen flux (mol/m^2^/sec). Note high oxygen concentration at air-liquid interface at the top of the bioreactor. Ramping up stirring speed creates a driving force for increasing oxygen content in the bioreactor. (**B**) Increase in oxygen content after initiation of stirring. Note that 5s of stirring at 140 rpm is expected to result in a twofold higher concentration of oxygen compared to 70 rpm.

**A**

**B**


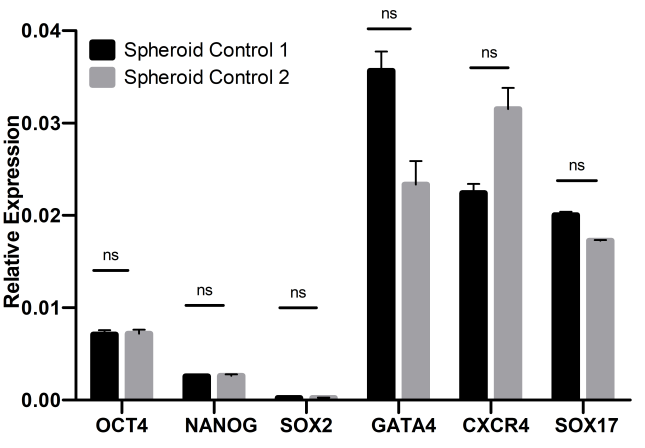

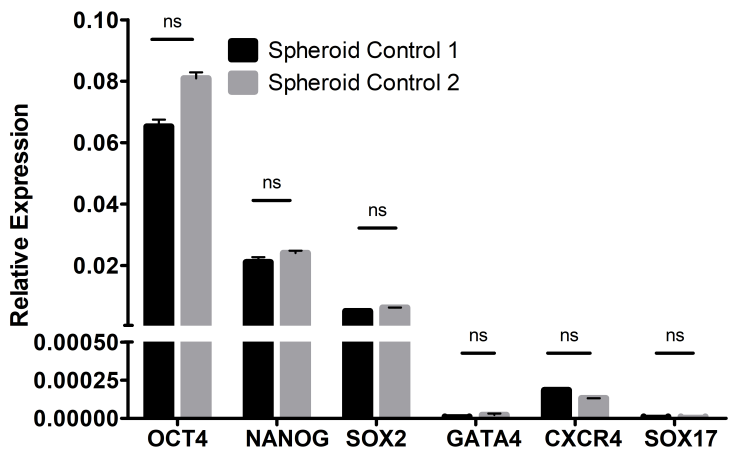


**Figure S6.** **Comparing gene expression for spheroid controls without capsules.** (A) RT-PCR analysis of gene expression after 3 days in pluripotency media. (B) Gene expression analysis after additional 3 days in endodermal differentiation media. Control 1 – hPSCs exposed to chemicals (high MW PEG and densifier) used during encapsulation process and then formed into spheroids in a bioreactor. Control 2 – hPSCs directly formed into spheroids. For statistical analysis - n = 4; p < 0.05.
